# Machine learning prediction for COVID-19 disease severity at hospital admission

**DOI:** 10.1186/s12911-023-02132-4

**Published:** 2023-03-07

**Authors:** Ganesh Raman, Bilal Ashraf, Yusuf Kemal Demir, Corey D. Kershaw, Sreekanth Cheruku, Murat Atis, Ahsen Atis, Mustafa Atar, Weina Chen, Ibrahim Ibrahim, Taha Bat, Mutlu Mete

**Affiliations:** 1 Departments of Internal Medicine and Pediatrics, University of Texas Southwestern Medical Center, Dallas, TX 75390 USA; 2 Departments of Internal Medicine, University of Texas Southwestern Medical Center, Dallas, TX 75390 USA; 3 School of Data Science, University of North Carolina at Charlotte, Charlotte, NC USA; 4 Department of Internal Medicine, Division of Pulmonary and Critical Care Medicine, University of Texas Southwestern Medical Center, Dallas, TX 75390 USA; 5 Department of Anesthesiology and Pain Management, Divisions of Cardiothoracic Anesthesiology and Critical Care Medicine, University of Texas Southwestern Medical Center, Dallas, TX 75390 USA; 6 Department of Radiology, University of Texas Southwestern Medical Center, Dallas, TX 75390 USA; 7 Department of Internal Medicine, Division of Hematology and Oncology, University of Texas Southwestern Medical Center, Dallas, TX 75390 USA; 8 Cleveland Clinic, Cleveland, OH 44195 USA; 9 Department of Pathology, Hematopathology Section, University of Texas Southwestern Medical Center, Dallas, TX 75390 USA; 10 Department of Computer Science and Information Systems, Texas A&M University – Commerce, Commerce, TX 75429-3011 USA

**Keywords:** Laboratory markers, COVID-19, SARS-CoV-2, Prediction, Scoring, Machine learning, Classification

## Abstract

**Importance:**

Early prognostication of patients hospitalized with COVID-19 who may require mechanical ventilation and have worse outcomes within 30 days of admission is useful for delivering appropriate clinical care and optimizing resource allocation.

**Objective:**

To develop machine learning models to predict COVID-19 severity at the time of the hospital admission based on a single institution data.

**Design, setting, and participants:**

We established a retrospective cohort of patients with COVID-19 from University of Texas Southwestern Medical Center from May 2020 to March 2022. Easily accessible objective markers including basic laboratory variables and initial respiratory status were assessed using Random Forest’s feature importance score to create a predictive risk score. Twenty-five significant variables were identified to be used in classification models. The best predictive models were selected with repeated tenfold cross-validation methods.

**Main outcomes and measures:**

Among patients with COVID-19 admitted to the hospital, severity was defined by 30-day mortality (30DM) rates and need for mechanical ventilation.

**Results:**

This was a large, single institution COVID-19 cohort including total of 1795 patients. The average age was 59.7 years old with diverse heterogeneity. 236 (13%) required mechanical ventilation and 156 patients (8.6%) died within 30 days of hospitalization. Predictive accuracy of each predictive model was validated with the 10-CV method. Random Forest classifier for 30DM model had 192 sub-trees, and obtained 0.72 sensitivity and 0.78 specificity, and 0.82 AUC. The model used to predict MV has 64 sub-trees and returned obtained 0.75 sensitivity and 0.75 specificity, and 0.81 AUC. Our scoring tool can be accessed at https://faculty.tamuc.edu/mmete/covid-risk.html.

**Conclusions and relevance:**

In this study, we developed a risk score based on objective variables of COVID-19 patients within six hours of admission to the hospital, therefore helping predict a patient's risk of developing critical illness secondary to COVID-19.

## Introduction

The COVID-19 pandemic began as an outbreak of the SARS-CoV2 virus in the Wuhan province in China in December 2019. As of July 2022, there have been over 547,000,000 confirmed cases of COVID-19 worldwide [1]. The illness manifests itself variably, ranging from mild viral symptoms, including fever, cough, congestion, and sore throat to life-threatening illness defined by sepsis, respiratory failure, venous thromboembolism (VTE), shock, and death [2]. As COVID-19 is likely to continue affecting populations across the world, with novel virulent strains regularly emerging, it is critical to determine which patients are at risk for the more severe manifestations. While developed nations are more prepared for larger spikes associated with these emerging new variants, developing counties may still be at risk for shortages of resources, such as hospital and ICU beds [8]. These countries would uniquely benefit from newer, more robust COVID-19 scoring tools to best allocate their limited resources.

Artificial intelligence (AI) models have been developed to assist with COVID-19 risk stratification methods that use electronic health records (EHR) and laboratory results. These models use various types of data, such as demographic information, disease history, laboratory results, and clinical symptoms, to predict the likelihood of a patient developing severe COVID-19. In a retrospective study with 3988 patients, Grasselli et al. [3] reported that the survival rate of critically ill patients with COVID-19, particularly older men who require noninvasive mechanical ventilation and have preexisting comorbidities, is low. Hypertension was the most common comorbidity among patients, and those with hypertension had a significantly lower survival rate. Another study [4] investigated 4997 patients and performed a retrospective review of medical records of demographics, comorbidities and laboratory tests at the initial presentation of patients to develop a prediction model and risk scores of ICU admission and mortality in COVID-19. Similar to our research, they set ICU admission and death as the primary outcomes. The top five predictor reported as lactate dehydrogenase (LDH), procalcitonin, smoking history, oxygen saturation (SpO2), and lymphocyte count. Initially the use of lab values, such as c-reactive peptide (CRP) or d-dimer aided clinical decision-making in conjunction with clinical findings. The novel marker of immature platelet fraction % (IPF%) has been shown to be a predictor of clinical outcomes in COVID-19 [5, 6]. In our prior study, IPF% was predictive of hospital length of stay and intensive care unit (ICU) admission, the two crucial outcomes needed to help determine resource allocation. Since then, a variety of COVID-19 scoring tools have supplanted the original, more limited methods. These earlier scoring tools have a couple limitations. First, many have bias for high-risk patients [7]. Second, different virus variants, such as delta and omicron, were not integrated in the model.

The development of newer COVID-19 scoring tools should consider novel evidence and objective biomarkers of disease. Furthermore, this ideal scoring system should use admission standard laboratory values to be feasible in any clinical setting. This study aims to utilize an artificial intelligence (AI) method to predict severity of illness of COVID-19 patients by using initially obtained laboratory values, helping clinicians to identify patients at risk for disease progression, morbidity, and mortality.

## Methods

This study describes SARS-COV-2-infected patients evaluated at the University of Texas Southwestern Medical Center (UTSW) between May 2020 and March 2022. Patients were enrolled from the institutional COVID-19 patient registry, a local institutional review board approved registry comprised of COVID-19 patients designed to study the natural history of the disease. The current study included 1,795 adult patients with SARS-COV-2 infection (captured all major variants seen at Texas state) who also had IPF% measurements (Table 1).

**Table 1:** Patient demographics

	Characteristic		All (N = 1795)	30D mortality		Mech ventilation	
				Yes (n = 156)	No (n = 1639)	Yes (n = 236)	No (n = 1559)
1	Age	Years; mean, SD	59.7 ± 16.6	67.2 ± 14.5	59 ± 16.6	59.8 ± 14.9	59.6 ± 16.8
2	BMI	kg/m^2^	31.4 ± 8.3	30.3 ± 9.2	31.5 ± 8.2	32 ± 9.2	31.3 ± 8.1
3	Covid Vac. Adm	Yes/No	809	32	777	69	740
4	WBC	10^9^/L	8.2 ± 9.9	9.7 ± 12.3	8 ± 9.6	9.8 ± 10.4	7.9 ± 9.8
5	Absolute Neutrophils Count	1000/mm^3^	6.1 ± 4.8	7.5 ± 5	6 ± 4.7	7.7 ± 4.8	5.9 ± 4.7
6	Mean Corpuscular Volume	Femtoliters (fL)	87.9 ± 7.6	90.6 ± 7	87.6 ± 7.6	88.9 ± 6.6	87.7 ± 7.7
7	Platelet Count	10^9^/L	223.9 ± 100.8	179.1 ± 91	228.2 ± 100.6	204.5 ± 97.6	226.8 ± 101
8	Ferritin	ng/mL	1247.1 ± 3680	2969.4 ± 10,573.8	1083.2 ± 1985.7	2621 ± 8694	1040 ± 1978
9	Troponin I HS	ng/L	109 ± 945.3	371.7 ± 1931	84 ± 786.6	319 ± 2133	77.5 ± 581.3
10	AST	U/L	64.6 ± 167.2	114.9 ± 356.4	59.8 ± 135.4	106 ± 350.2	58.4 ± 116
11	ALT	U/L	47 ± 120.2	59.4 ± 140.6	45.8 ± 118.1	66.3 ± 221.6	44.1 ± 95.9
12	Albumin	g/dL	3.6 ± 0.5	3.4 ± 0.5	3.6 ± 0.5	3.4 ± 0.5	3.6 ± 0.4
13	Creatinine	mg/dL	1.6 ± 2.2	1.9 ± 2	1.5 ± 2.2	1.5 ± 1.5	1.6 ± 2.3
14	D-DIMER	mg/dL	2.2 ± 4.4	4.1 ± 8	2.1 ± 3.8	3.8 ± 7.3	2 ± 3.7
15	CRP	mg/dL	90.2 ± 73.9	120.3 ± 81.5	87.4 ± 72.5	131 ± 87.7	84.1 ± 69.6
16	Immature Platelet Fraction	Percentage	4.9 ± 5	5.3 ± 2.6	4.9 ± 5.1	5.6 ± 3	4.8 ± 5.2
17	BUN	mg/dL	21.8 ± 17.1	31.2 ± 21	20.9 ± 16.4	26.4 ± 16.6	21.1 ± 17.1
18	Initial O^2^ Flow Rate	L/min	4.8 ± 6.6	7.9 ± 12.3	4.5 ± 5.7	9 ± 12.4	4.2 ± 4.9
19	Lowest O^2^ Flow Rate within 4 h	L/min	3.8 ± 5.3	6.7 ± 11.2	3.5 ± 4.2	6.5 ± 9.7	3.3 ± 4.1
20	Highest O^2^ Flow Rate within 4 h	L/min	6.8 ± 10.3	13 ± 17.2	6.2 ± 9.2	14.9 ± 18.6	5.6 ± 7.7
21	Initial Respiratory Rate	L/min	19.8 ± 3.9	21.5 ± 5.8	19.6 ± 3.7	21.9 ± 6.1	19.5 ± 3.4
22	Lowest Resp. Rate within 4 h		16.7 ± 3.1	17 ± 3.3	16.7 ± 3.1	17.6 ± 3.8	16.6 ± 2.9
23	Highest Resp. Rate within 4 h		26.1 ± 6.8	28.9 ± 9	25.8 ± 6.5	30.1 ± 9.1	25.5 ± 6.1
24	Initial SpO^2^	Percentage	93.2 ± 6.5	89.6 ± 9.6	93.6 ± 6	87.7 ± 12	94.1 ± 4.7
25	Lowest Pulse oximetry within 4 h	Percentage	90.6 ± 6.8	86.9 ± 10.4	90.9 ± 6.2	85 ± 12.4	91.4 ± 5

Two predictive models were designed: one for 30-day mortality (30DM) (n = 156) and one for mechanical ventilation (n = 236). For these models, 120 demographic and pretreatment variables were collected. From this group, 25 variables (Table 2) were selected using Random Forest’s feature importance score. Selection of the optimal subset of 25 variables for the best-performing prediction model requires an exhaustive search and is computationally prohibitive. Random Forest (RF) algorithms are run repeatedly with different random settings and parameter sets to make sure significant variables rank higher in average during this iterative process. The domain experts approved the list of variables for the prediction task based on (1) clinical relevance when deciding mortality and mechanical ventilation risks, and (2) availability in emergent admission (hence, practically used in an early decision-making tool). Using weighted averages from feature importance rankings, we used a unified list of variables in both predictive models. Note that experimental accuracies using separate lists of variables for each model do not improve significantly.

**Table 2:** Variable correlations and feature rankings

	Sorted features (w/RF)	Correlation Coefficient		Average variables significance
		30DMort	Mec. Vent	
1	Highest O^2^ Flow Rate within 4 h	0.186	0.304	3.316
2	Initial SpO^2^	− 0.173	− 0.328	3.085
3	Lowest Pulse Oximetry within 4 h	− 0.168	− 0.318	3.082
4	Lowes O^2^ Flow Rate within 4 h	0.175	0.203	2.692
5	Ferritin	0.144	0.145	2.604
6	CRP	0.125	0.214	2.466
7	Initial O^2^ Flow Rate	0.141	0.246	2.432
8	BUN	0.169	0.105	2.414
9	Highest Respiratory rate within 4 h	0.127	0.231	2.347
10	AST	0.093	0.096	2.345
11	Albumin	− 0.167	− 0.179	2.281
12	Platelet Count	− 0.137	− 0.075	2.202
13	Absolute Neutrophil count	0.085	0.131	2.180
14	Initial Respiratory Rate	0.132	0.212	2.159
15	Troponin I HS	0.086	0.086	1.964
16	WBC	0.049	0.064	1.919
17	Age	0.140	0.004	1.737
18	D-DIMER	0.132	0.135	1.720
19	Immature Platelet Fraction	0.020	0.055	1.633
20	Mean Corpuscular Volume	0.109	0.053	1.591
21	Creatinine	0.042	− 0.011	1.502
22	BMI	N/A*	N/A*	1.358
23	Lowest Respiratory rate within 4 h	0.023	0.109	1.247
24	COVID-19 Vaccination Administration	0.152	0.126	1.243
25	ALT	0.032	0.062	1.238

The prediction task entails correct identification of patients at risk of mortality within 30 days of admission. The significant advantages of an RF model include measurability of variable importance for prediction, handling of a mixture of numerical and categorical variables, and accuracy that is comparable to other prominent methods [9, 10]. Random Forest is an ensemble method that crowdsources predictions from multiple trained decision trees for a more accurate prediction. A decision tree, the constituent machine learning algorithm in an RF framework, produces the probability of a class by hierarchically splitting nodes based on independent variables into buckets of values (e.g., a split could be “Platelet Count” < 145 10^9^/L) until a leaf node with a class label prediction is reached. The collection of splits used in reaching the leaf node constitutes the rule for a final probability assessment of the outcome variable, the prediction. The choice and order of nodes and splits used in a decision tree led to variation in the collection of rules, the associated prediction, and the overall performance.

The RF model was trained within an open-source software kit, Scikit-learn [11], to identify 30DM and ventilation candidates at risk. Because the training of an RF requires first determining the number of iterations (i.e., number of embedded decision trees), number of randomly selected variables in each tree, and the depth of the decision trees (e.g., the number of splits), an optimization approach and performance validation is required to produce the final model to obtain the best performance. Our final RF model was optimized over a prediction search space of multiple parameters. The search space optimization aimed to achieve higher and lower performance bounds on sensitivity (≥ 0.75) and specificity (≥ 0.70), respectively. The relatively larger lower bound on specificity may permit higher false positive predictions resulting in unwanted predictions. However, the inverse approach, misclassification of patients at risk, would mean a higher mortality or misallocation of ventilation rooms. After an initial parameter tuning, the search was performed by selecting the best-performing model by training RFs with 64 to 256 decision trees (in increments of 32), 10 to 30 predictor variables, 1 to 5 features to consider when looking for the best split, 3 to 9 tree height (in increments of 1) and using Gini index as a splitting criterion [9]. Because the data has a low positive rate (8.6% for 30DM, and 13% for ventilated patients), known as unbalanced classification, more weight is assigned to the positive class. In the ensemble step, models with high accuracy were given more weight in deciding the final prediction. The feature importance score is calculated and reported for the final RF model variables.

Cross-validation was used in measuring the performance of models created when searching for the parameters of an optimal model. In this study, the standard ten-fold cross-validation was employed [12]. Under ten-fold cross-validation, the dataset was divided into ten non-overlapping cohorts, with each cohort having a similar proportion of positive subjects. Based on cross-validated test subjects, the area under the curve (AUC) for Receiver Operating Characteristics (ROC) or c-statistic was calculated. Our prediction model assumes that all variables are presented for each patient. One exception is made for IPF since it is not a routinely measured lab value. If an IPF value is missing, it is predicted based on the patient's other lab results and demographic data using the K-nearest neighbors method (K = 50) in the Scikit-learn library [11].

## Results

Of a total of 1795 patients, 52.6% were males. The average age was 59.7 years old. 38.3% of the study cohort were white, 30.7% were black, 24.0% were Hispanic. 58.6% of the patients had existing hypertension, while 38.2% had diabetes mellitus type 2. Of the 1795 hospitalized patients, 236 (13%) required mechanical ventilation and 156 patients (8.6%) died within 30 days of hospitalization.

**Fig. 1:**
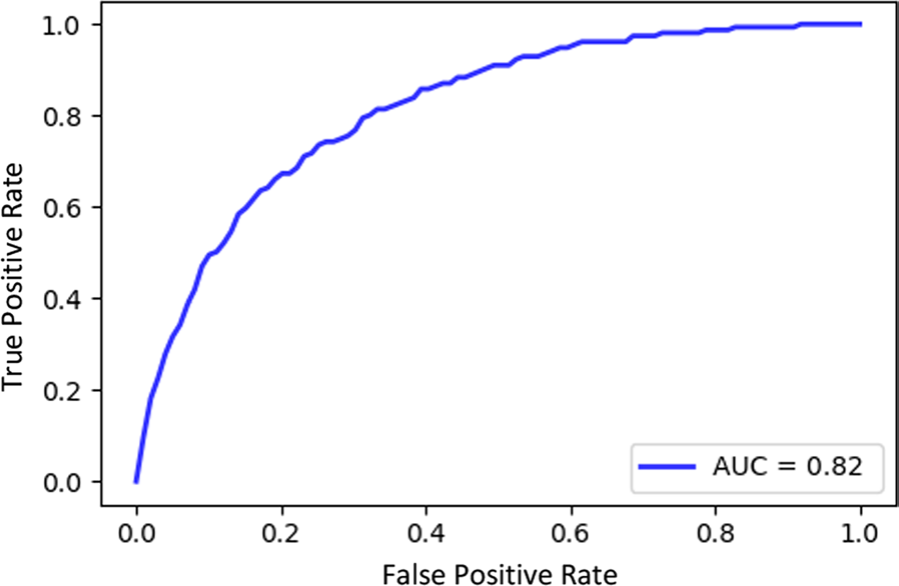
C-statistic for 30DM

**Fig. 2:**
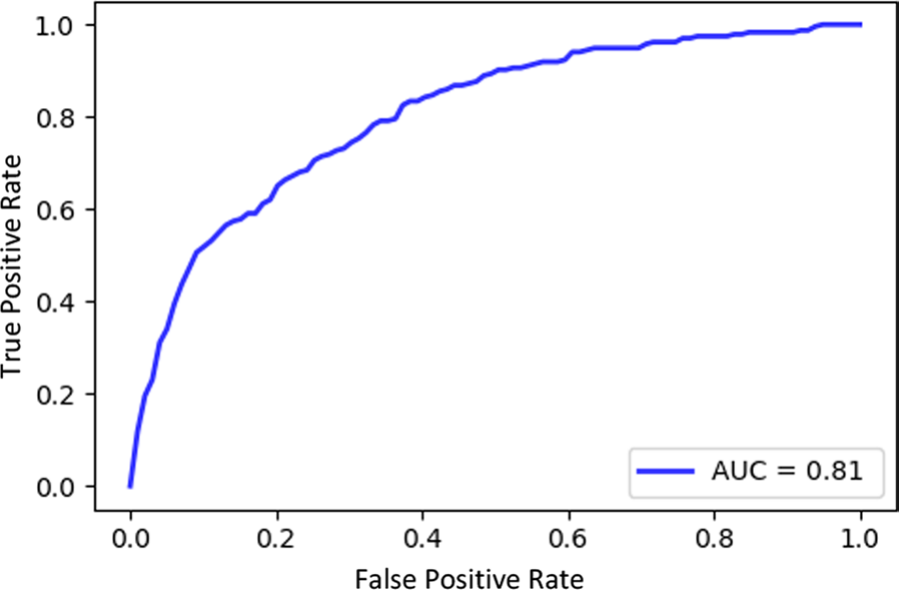
C-statistic for ventilation

Predictive accuracy of each predictive model is validated with the 10-CV method. Random Forest classifier for 30DM model has 192 sub-trees, and obtained 0.72 sensitivity and 0.78 specificity, and 0.82 AUC. The model used to predict MV has 64 sub-trees and returned obtained 0.75 sensitivity and 0.75 specificity, and 0.81 AUC. Tables 1 and 2 summarize important scores and correlation coefficients for each variable used in predictions. Figures 1 and 2 displays AUCs for 30DM and mechanical ventilation predictions, respectively.

We were then able to assign categories to score ranges. For each subject, the classifiers assign a score between 0 and 1 with a positive/negative threshold of 0.5. The higher the score, the more severe the risk. A negative subject with a score of 0.35 is healthier than a subject with 0.47, for example. Likewise, a subject with 0.75 poses higher risks than another positive subject who received a score of 0.67. We categorized each negative subject (0.0–0.49) as “insignificant risk.” Positive range of 0.50 to 1.00 categorized as (a) low risk for scores from 0.5 to 0. 65, (b) moderate risk for 0.66–0.80, and (c) high risk for 0.81–1.00. In cross-validation experiments, 30D mortality predictor identified 352 low risk, 122 moderate risk, and 15 high risk subjects. The risk predictor for mechanical ventilation returned 456 low risk, 153 moderate risk, and 24 high risk subjects in the dataset. Our scoring tool can be accessed at https://faculty.tamuc.edu/mmete/covid-risk.html.

## Discussion

Resource allocation has been a major challenge for all healthcare facilities. Laboratory values shown to be predictive of COVID-19 severity have been identified, and their feasibility of utilization is varied [13]. Existing scoring systems using more subjective and less objective markers predicting severity and outcomes in COVID‐19 patients need to be improved to mitigate clinician’s subjectivity and provide quick severity assessment within six hours of admission.

In our current study of a large, hospitalized cohort of COVID‐19 patients, we have found 25 markers to be effective in predicting COVID-19 severity of illness via 30DM and need for mechanical ventilation by using AI technology.

The advantages of our model include classification of scores into easy-to-understand risk categories. This allows easy application for allocation decisions in situations of resource shortages, when a patient’s severity of illness or predicted outcome determines where said resources must be deployed to prevent poor outcomes. Categories can also be used to determine the nature of post-discharge care and follow up. There are also potential research applications, as these risk stratification categories can be used to identify patients for focused clinical trials. Further, our prediction models easily can categorize patients from readily available objective variables which can be utilized at the time of the early admission. Our scoring tool does use novel IPF, and although this is not a commonly obtained test, it can be added on to a regular complete blood count in peripheral blood samples. We have also incorporated vaccination status which earlier scoring tools have not used [14]. Though the AUC score (predictive value) is higher than many of the other COVID-19 scoring models [15], the advantage of our score is in the inclusion of a larger number of prognosticating markers compared to other tools as well as a larger and more diverse patient database with all major COVID-19 variants. These strengths allow for a greater degree of generalizability to populations within the United States [14, 16].

Our model could be strengthened further with the addition of imaging data, such as chest x-rays or computed tomography scans [14]. Additionally, with vaccination status there are differences in rates of breakthrough infections between commercially available vaccines, and between patients at various stages of their vaccination series (partially vaccinated versus fully vaccinated versus boosted) [17].

## Conclusion

By using AI, we identified 25 prognostic markers that were significantly associated with 30DM and mechanical ventilation in hospitalized COVID-19 patients. Our scoring system using these markers showed 0.75 sensitivity and 0.75 specificity, and 0.81 AUC in predicting our primary outcomes. This score offers a novel way of prognosticating hospitalized COVID-19 patients by risk category in the United States and is therefore helpful for resource allocation and anticipation of the level of care these patients will need.

## Data Availability

The data can be made available upon reasonable request from the corresponding author.
